# Supplementary material for: Enhanced Anti-Skin-Aging Activity of Yeast Extract-Treated Resveratrol Rice DJ526
Source: Molecules. 2022 Mar 17;27(6):1951. doi: 10.3390/molecules27061951 (PMC8954687; doi:10.3390/molecules27061951)

Supplementary information

Table S1 Primer sequences used for real-time PCR experiments

	Genes	Sequences
1	GAPDH	Forward 5′-AACTTTGGCATTGTGGAAGG-3′<br>Reverse 5′-ACACATTGGGGGTAGGAACA
2	MITF	Forward 5′-TGAAGGTCGGTGTGAACGGATTTCGC-3′<br>Reverse 5′-CATGTAGGCCATGAGGTCCACCAC-3′
3	Tyrosinase	Forward 5′-GACGGTCACTGCAGACTTTG-3′<br>Reverse 5′-GCCATGACCAGGATGAC-3′
4	TRP-1	Forward 5′-ACTTCACTCAAGCCAACTGC 3′<br>Reverse 5′-AGCTTCCCATCAGATGTCGT 3′
5	TRP-2	Forward 5′GCTCCAAGTGGCTGTAGACC 3′<br>Reverse 5′ AATGCAGTGGCTTGGAAATC 3′
6	HPRT1	Forward 5′ TGCTCGAGATGTGATGAAGG 3′<br>Reverse 5′ TCCCCTGTTGACTGGTCATT 3′
6	TNF-α	Forward 5′ CAGAGGGCCTGTACCTCATC 3′<br>Reverse 5′ GGAAGACCCCTCCCAGATAG 3′
7	MMP-2	Forward 5′ GCTGTATGTCCTGTCGCTCA 3′<br>Reverse 5′ TGCTGAAAGAAATGCACACC 3′
8	MMP-9	Forward 5′ TCTTCCCTCCCATCAGTTTG 3′<br>Reverse 5′ GGACACCAGACCAAGGAAGA 3′
9	MMP-13	Forward 5′ CTTCCTGGTCAACCTCTCCA 3′<br>Reverse 5′ CCAATTGGCACCTTCTCTTT 3′
10	COL1A1	Forward 5′ GCAAGATGGAGTCAGGGAAA 3′<br>Reverse 5′ AGCCAGCAGATCGAGAACAT 3′
11	Filaggrin	Forward 5′ CCGATATCAGGACACAAGCA 3′<br>Reverse 5′ TGTTTCTCTTGGGCTCTTGG3′

Figure S1 HPLC chromatogram analysis of total resveratrol product (resveratrol and piceid content) of methanolic DJ 526 and yeast extract-treated DJ526 seed extracts. Piceid presents at retention time 16.8 min. and resveratrol presents at 27.6 min.

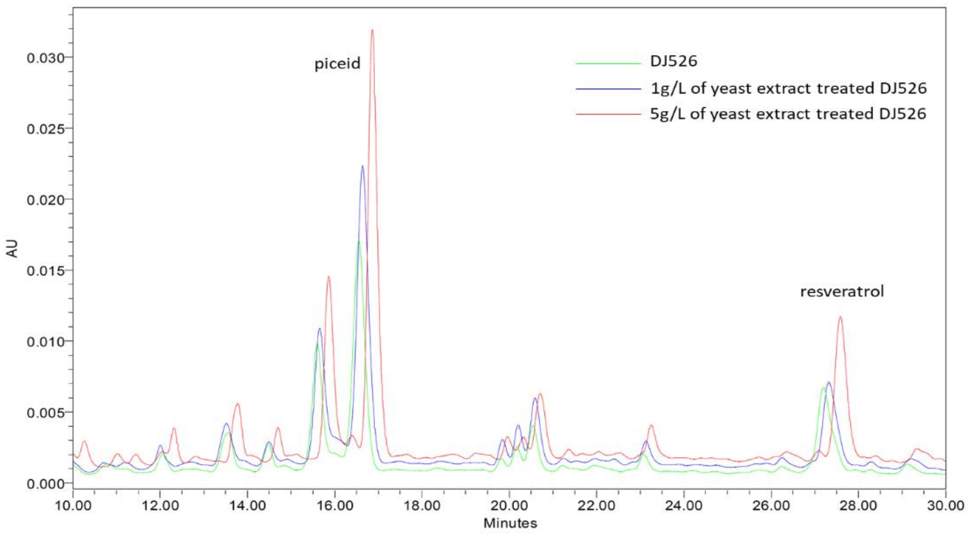

Figure S2 Calibration curve of vitamin C at concentration 0.0039065 to 0.25 mg/mL.

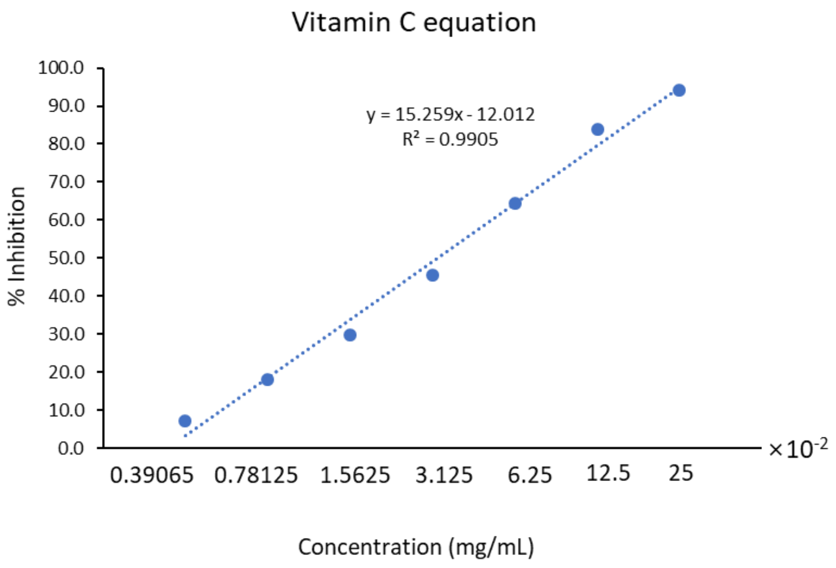

Supplement: Supplementary file 1 [file molecules-27-01951-s001.zip › molecules-1618743-supplementary.pdf]
